# Validation of the Dudley inflammatory bowel symptom questionnaire for assessing gastrointestinal symptom burden in axial spondyloarthritis

**DOI:** 10.1007/s00296-026-06108-1

**Published:** 2026-04-09

**Authors:** Meryem Ozoglu, Duygu Temiz Karadag, Neslihan Gokcen, Ozlem Ozdemir Isik, Hasan Yilmaz, Ayse Cefle, Ayten Yazici

**Affiliations:** 1 Department of Internal Medicine, School of Medicine, Kocaeli, Turkey; 2 Department of Internal Medicine, Division of Rheumatology, Kocaeli University, School of Medicine, Kocaeli, Turkey; 3 Department of Internal Medicine, Division of Gastroenterology, Kocaeli University, School of Medicine, Kocaeli, Turkey

**Keywords:** Axial spondyloarthritis, Inflammatory bowel disease, Dudley inflammatory bowel symptom questionnaire, Questionnaire

## Abstract

Subclinical and overt inflammatory bowel disease (IBD) is frequently observed in patients with axial spondyloarthritis (ax-SpA), traditionally identified through invasive ileocolonoscopic evaluation. Validated non-invasive assessing instruments are therefore needed to facilitate early detection. This study aimed to validate the Turkish version of the Dudley Inflammatory Bowel Symptom Questionnaire (DISQ) as a tool for assessing gastrointestinal symptom burden suggestive of intestinal involvement in patients with axial spondyloarthritis. In this cross-sectional validation study, 174 patients fulfilling the 2009 Assessment of SpondyloArthritis international Society (ASAS) criteria for axial SpA were enrolled. Convergent validity was assessed by examining correlations between DISQ scores and disease activity (Bath Ankylosing Spondylitis Disease Activity Index [BASDAI]), functional status (Bath Ankylosing Spondylitis Functional Index [BASFI]), and health-related quality of life (Short Form-36 [SF-36]). Discriminant validity was evaluated by comparing DISQ scores in patients with and without gastrointestinal symptoms suggestive of IBD. Test–retest reliability was assessed by re-administration of the DISQ after one month. Internal consistency was evaluated using Cronbach’s alpha. DISQ scores demonstrated moderate positive correlations with BASDAI (r = 0.432, p < 0.001) and BASFI (r = 0.248, p = 0.001), and a strong inverse correlation with SF-36 scores (p < 0.001), supporting convergent validity. Patients reporting diarrhea, abdominal pain, oral aphthae, or mucus in stool had significantly higher DISQ scores than those without these symptoms (all p ≤ 0.008), confirming discriminant validity. The Turkish version of the DISQ demonstrated acceptable internal consistency (Cronbach’s α = 0.70) and good test–retest reliability (ICC = 0.76; 95% CI: 0.51–0.82; p < 0.001). The Turkish version of DISQ demonstrates satisfactory validity and reliability as a non-invasive assessment instrument for gastrointestinal symptoms suggestive of bowel inflammation in patients with ax-SpA. Future studies incorporating endoscopic and biomarker-based outcomes are warranted to define clinically meaningful cut-off values and determine its role in routine clinical practice.

**Supplementary Information:**

The online version contains supplementary material available at 10.1007/s00296-026-06108-1.

## Introduction

Spondyloarthritis (SpA) encompasses a group of interrelated inflammatory rheumatic diseases that may affect the axial skeleton (axial spondyloarthritis [ax-SpA]) and/or peripheral joints (peripheral SpA), with ankylosing spondylitis (AS) representing the prototypical form. Beyond musculoskeletal manifestations, SpA is frequently associated with extra-articular features, most commonly inflammatory bowel disease (IBD), uveitis, and psoriasis [1–3].

Intestinal inflammation is common in patients with SpA. Ileocolonoscopic studies have shown inflammatory bowel lesions in up to 60% of patients with AS, underscoring the close association between SpA and intestinal involvement. SpA is increasingly recognized as a spectrum of genetically related conditions that share pathogenic pathways with IBD. Clinical, histopathological, and immunological evidence indicates that mechanisms central to IBD, particularly intestinal immune dysregulation and loss of tolerance to commensal microbiota, also play a key role in ax-SpA [4–7].

Despite the high prevalence of subclinical intestinal inflammation, clinically overt IBD is observed in fewer than 15% of patients with AS [6–9]. Longitudinal studies suggest that a subset of patients with subclinical intestinal inflammation subsequently develop Crohn’s disease, with reported progression rates ranging from 6.5% to 20% within five years [10]. Recent large-scale cohort studies have confirmed that patients with ankylosing spondylitis have a substantially increased risk of developing inflammatory bowel disease compared with both psoriasis and the general population, supporting the concept of a shared gut–joint inflammatory axis and emphasizing the clinical relevance of identifying gastrointestinal symptoms in patients with axial spondyloarthritis [11]. Furthermore, microscopic intestinal inflammation has been described in patients with spondyloarthritis and is associated with younger age, male sex, earlier disease onset, higher disease activity, radiographic sacroiliitis, and reduced spinal mobility [12]. Consistent with these observations, a 2015 meta-analysis estimated the pooled prevalence of clinically overt IBD in AS at 6.8%, while more recent registry-based studies have reported comparable prevalence estimates of approximately 5%, confirming that a clinically meaningful minority of patients within the SpA spectrum have coexisting IBD [8, 13].

Avoiding diagnostic delay is crucial for both SpA and IBD, as delays are associated with worse clinical outcomes and poorer treatment response [8, 14]. Early identification of these conditions is essential to ensure that patients benefit from timely and appropriate treatment during the initial stages of the disease. The Dudley Inflammatory Bowel Symptom Questionnaire (DISQ) is a validated, patient-reported outcome tool designed to identify symptoms suggestive of intestinal inflammation [15, 16]. It evaluates various gastrointestinal complaints, including diarrhea, abdominal pain, mucus in stool, and rectal bleeding, which are often associated with IBD. The DISQ provides a non-invasive, systematic method for assessing patients at risk of IBD, enabling clinicians to identify candidates for further diagnostic evaluation. Its simplicity and accessibility make it a practical choice for use in routine clinical practice.

Although the DISQ has been validated for use in axial spondyloarthritis, a Turkish-language version had not been previously validated [16]. By integrating clinical and laboratory screening parameters, the present study aims to streamline the identification of patients who warrant gastroenterologic evaluation and to support earlier recognition of intestinal involvement in spondyloarthritis.

## Materials and methods

###  Study Population

This cross-sectional study included 174 patients who met the Assessment of SpondyloArthritis international Society (ASAS) Ax-SpA criteria and were followed up at the Rheumatology Clinic [12], department of internal medicine, Kocaeli University Faculty of Medicine. Consecutive patients attending the rheumatology outpatient clinic between [12/2021–09/2022] were screened for eligibility.

Patients aged between 18 and 75 years with a diagnosis of ax-SpA, who were fluent in Turkish and able to understand written information, were included in the study. Patients with enteropathic arthritis, psoriatic arthritis, other inflammatory rheumatic diseases, established inflammatory bowel disease, malignancy, active systemic infection, cognitive impairment, or insufficient proficiency in Turkish were excluded.

Demographic and clinical data were extracted from outpatient clinic records and included age, sex, disease duration, age at diagnosis, comorbidities, medication use (type and duration), and HLA-B27 status. Laboratory parameters comprised C-reactive protein, erythrocyte sedimentation rate, albumin, globulin, liver and renal function tests, complete blood count, ferritin, vitamin B12, and iron studies. Disease activity and functional status were assessed using the Bath Ankylosing Spondylitis Disease Activity Index (BASDAI) and the Bath Ankylosing Spondylitis Functional Index (BASFI). Available imaging findings were recorded. Extra-intestinal manifestations suggestive of inflammatory bowel disease were documented when present.

Gastrointestinal symptoms were evaluated using a structured clinical interview based on established IBD alarm features. The presence of diarrhea, abdominal pain, weight loss, rectal bleeding, blood in stool, mucus in stool, and perianal disease (including fistula, fissure, abscess, skin tags, or perianal maceration) was recorded. Stool characteristics were assessed using the Bristol Stool Form Chart (BSFC), and stool types were documented according to the seven-category BSFC classification. In addition, extraintestinal manifestations associated with IBD, including erythema nodosum, pyoderma gangrenosum, oral aphthous ulcers, and cholangitis, as well as family history of inflammatory bowel disease, were documented when present.

This study was approved by the Non-Interventional Clinical Research Ethics Committee of Kocaeli University (Date: November 18, 2021; Decision No: KÜ GOKAEK-2021/20.13). The study protocol adhered to the principles of the Declaration of Helsinki and Good Clinical Practice guidelines. Written informed consent was obtained from all participants after providing detailed information about the study.

### Definitions of the scales

*Dudley Inflammatory Bowel Symptom Questionnaire (DISQ):* The DISQ is a symptom-based questionnaire originally developed to assess bowel symptoms in patients with inflammatory bowel disease and later applied in studies of patients with spondyloarthritis [15]. The modified version used in this study consists of 15 questions, each scored on a five-point numerical scale (0 = none/never, 1 = mild/sometimes, 2 = moderate, 3 = severe, 4 = very severe) (Table 1) [16]. The questions are divided into two broad categories: bowel symptoms and systemic symptoms. The total DISQ score ranges from 0 to 60, with higher scores indicating more severe symptoms. The questionnaire is self-administered. A score ≥ 11 has been suggested to indicate clinically relevant bowel symptoms affecting quality of life [15, 16]. The Turkish version of the DISQ used in this study is provided in Supplementary Table S1.

**Table 1:** Dudley Inflammatory bowel symptom questionnaire (DISQ)

DISQ questions	Scoring (0–4)
1. Stool frequency	0–4
2. Diarrhea	0–4
3. Blood in stool	0–4
4. Nighttime defecation	0–4
5. Urgency for defecation	0–4
6. Persistent sensation of incomplete defecation	0–4
7. Straining during defecation	0–4
8. Accidental soiling	0–4
9. Anal itching or pain	0–4
10. Gas incontinence	0–4
11. Abdominal cramps or pain	0–4
12. Appetite loss	0–4
13. Nausea	0–4
14. Fatigue	0–4
15. Fever	0–4

(0 = none/never, 1 = mild/sometimes, 2 = moderate, 3 = severe, 4 = very severe)

*Short Form Health Survey (SF-36):* SF-36 is a validated self-report questionnaire consisting of 36 multiple-choice, Likert-scale items. It was developed to assess the quality of life in patients with physical illnesses. The survey evaluates both positive and negative aspects of health over the past four weeks. It covers eight categories of health status: physical functioning, role limitations, social functioning, mental health, vitality, pain, general health perception, and emotional role limitations. The SF-36 is sensitive to differences in disability levels, with higher scores indicating better quality of life. The Turkish version of the SF-36 has been validated and successfully used in numerous studies on SpA [17].

*Bath Ankylosing Spondylitis Disease Activity Index (BASDAI):* BASDAI is a questionnaire designed to assess disease activity in AS. It consists of six questions addressing five key symptoms: fatigue, spinal pain, joint pain/swelling, localized tenderness, and morning stiffness. Patients are asked to rate the severity of these symptoms over the past week on a Visual Analog Scale (VAS) ranging from 0 to 10. The scores for the first four questions are summed, and the average of the two questions related to morning stiffness is added. The total score is then divided by 5, yielding a value between 0 and 10. Higher scores indicate active disease. A BASDAI score of ≥ 4 suggests disease activation, while a score of < 2 indicates stable disease. The Turkish version of BASDAI has been validated for reliability and validity in AS patients by Akkoc et al. [18].

### Validation of the psychometric properties of Turkish DISQ

All participants were asked to complete the Dudley Inflammatory Bowel Symptom Questionnaire (DISQ), the Short Form-36 Health Survey (SF-36), the Bath Ankylosing Spondylitis Disease Activity Index (BASDAI), and the Bath Ankylosing Spondylitis Functional Index (BASFI). Questionnaire administration was supervised by a trained investigator (M.O.), who provided standardized instructions to ensure adequate understanding.

*Translation and Cross-Cultural Adaptation:* The Turkish version of the DISQ was developed using a standardized forward–backward translation methodology in accordance with established guidelines for cross-cultural adaptation [19]. Briefly, the original English version of the DISQ was independently translated into Turkish by two translators, one with clinical familiarity with the questionnaire content and one without medical background. The translations were reconciled into a single consensus version by agreement between the translators and a study physician. This version was then back-translated into English by two native English speakers who were blinded to the original questionnaire. The back-translated versions were reviewed by an experienced gastroenterologist and a rheumatologist, and discrepancies were resolved by consensus. Following comparison with the original instrument and confirmation of conceptual equivalence, the final Turkish version of the DISQ was established.

*Comprehensibility Testing:* The comprehensibility of the Turkish version of the DISQ was assessed during its administration to patients. Participants were asked to report any difficulties in understanding the questionnaire items or response options. No major issues regarding clarity or comprehension were identified, and therefore no further modifications were required.

*Convergent validity:* Convergent validity was assessed by examining the associations between DISQ scores and measures of disease activity, functional status, and health-related quality of life, including BASDAI, BASFI, and SF-36 scores. We hypothesized that higher DISQ scores would correlate moderately with higher disease activity and functional impairment and inversely with quality-of-life measures, particularly the physical domains of the SF-36.

*Discriminant validity:* Discriminant validity was evaluated by comparing DISQ scores between patients with and without gastrointestinal symptoms suggestive of inflammatory bowel disease. We hypothesized that DISQ scores would be significantly higher among patients reporting gastrointestinal alarm features, supporting the instrument’s ability to distinguish between clinically relevant symptom groups.

*Test–Retest Reliability:* Test–retest reliability was assessed in a subgroup of 51 patients who completed the DISQ at baseline and again after a 4-week interval, provided that no major clinical change or treatment modification had occurred during this period. The questionnaire was re-administered during face-to-face interviews by the same investigator. Reliability was quantified using the intraclass correlation coefficient (ICC), with values ≥ 0.70 indicating good agreement [20].

*Internal Consistency:* Internal consistency of the Turkish DISQ was evaluated using Cronbach’s alpha, with values ≥ 0.70 considered acceptable for group-level comparisons.

### Laboratory evaluation

Laboratory parameters were obtained from the hospital laboratory database to characterize the clinical profile of the study population and to support disease activity assessment using routine inflammatory markers such as C-reactive protein (CRP) and erythrocyte sedimentation rate (ESR). These parameters were not used as primary measures in the validation of the questionnaire but were explored for potential associations with gastrointestinal symptom burden and disease activity.

### Statistical analysis

Descriptive statistics were used to summarize clinical and demographic characteristics. Categorical variables are presented as frequencies and percentages, and continuous variables as mean ± standard deviation (mean ± SD) or median (interquartile range [IQR = Q3–Q1]), as appropriate based on data distribution. The discriminative performance of DISQ scores relative to inflammatory bowel disease-related variables, disease activity indices, and laboratory parameters was evaluated using the Mann–Whitney U test or independent-samples t test for continuous variables, and the χ^2^ test for categorical variables, as appropriate. Associations between DISQ scores and measures of disease activity, functional status, and health-related quality of life (BASDAI, BASFI, and SF-36 scores) were assessed using Spearman’s rank correlation coefficients. Test–retest reliability was evaluated using the intraclass correlation coefficient (ICC). Correlation strength was interpreted as low (≤ 0.29), moderate (0.30–0.49), or high (≥ 0.50). All statistical analyses were conducted using SPSS version 20.0 (IBM Corp., Chicago, IL, USA). Two-sided p values < 0.05 were considered statistically significant.

## Results

### Patient characteristics

A total of 174 patients with axial spondyloarthritis were included in the study. The mean age was 44.5 ± 10.4 years; mean age at symptom onset and at diagnosis were 29.8 ± 9.0 and 34.3 ± 10.2 years, respectively, with a mean disease duration of 175.4 ± 104.3 months. Fifty-six patients (32.2%) were female.

All patients reported inflammatory back pain at the time of diagnosis, and 72 patients (41.4%) reported ongoing back pain at the time of assessment. Peripheral musculoskeletal manifestations during the disease course included arthritis in 28 patients (16.1%), dactylitis in 7 (4.0%), and enthesitis in 105 (60.3%). Extra-articular manifestations included uveitis in 27 patients (15.5%) and psoriasis in 7 (4.0%). A family history of spondyloarthritis was reported by 50 patients (28.7%). Most patients responded to nonsteroidal anti-inflammatory drugs (90.2%), and sacroiliitis was confirmed by magnetic resonance imaging in 127 of 135 evaluated patients (94.1%). Baseline demographic, clinical, imaging, and laboratory characteristics are summarized in Supplementary Table S2.

### Gastrointestinal and IBD-related clinical features

Clinical features potentially associated with inflammatory bowel disease were frequently reported. Diarrhea was present in 15 patients (8.6%), abdominal pain in 27 (15.5%), mucus in stool in 21 (12.1%), and perianal disease in 41 (23.6%). Rectal bleeding and visible blood in stool were reported by 3.4% and 4.0% of patients, respectively, while unexplained weight loss was uncommon (4.0%). A family history of inflammatory bowel disease was reported by three patients (1.7%). Extraintestinal manifestations potentially related to IBD included oral aphthous ulcers in 34 patients (19.5%), erythema nodosum in 4 (2.3%), and cholangitis in 1 patient (0.6%).

### Convergent validity

DISQ scores demonstrated a moderate positive correlation with disease activity as measured by BASDAI (r = 0.432, p < 0.001) and a weaker but statistically significant correlation with functional impairment assessed by BASFI (r = 0.248, p < 0.001). DISQ scores were inversely correlated with all domains of the SF-36, with the strongest associations observed for bodily pain, social functioning, and vitality (all p < 0.001), supporting convergent validity (Table 2).

**Table 2:** Correlation of DISQ with SF-36, BASDAI, and BASFI

DISQ	r	p
SF-36		
Physical function*	− 0.342	(< 0.001)
Physical role limitation*	− 0.332	(< 0.001)
Emotional role limitation*	− 0.310	(< 0.001)
Energy/vitality*	− 0.384	(< 0.001)
Mental health*	− 0.363	(< 0.001)
Social functioning*	− 0.426	(< 0.001)
Pain*	− 0.468	(< 0.001)
General health perception*	− 0.470	(< 0.001)
BASDAI	0.432	(< 0.001)
BASFI	0.248	(< 0.001)

^*^Sub-parameters of the Short Form-36 Health Survey

*BASDAI* Bath ankylosing spondylitis disease activity index, *BASFI* Bath ankylosing spondylitis functional index, *DISQ* Dudley inflammatory bowel symptom questionnaire

### Discriminant validity

DISQ scores were significantly higher in patients reporting diarrhea, abdominal pain, oral aphthous ulcers, mucus in stool, and high disease activity (BASDAI ≥ 4) compared with those without these features (all p ≤ 0.008). In contrast, DISQ scores did not differ significantly according to the presence of perianal disease, anemia, or iron-deficiency anemia (Table 3).

**Table 3:** Discriminative ability of DISQ scores based on IBD-related parameters, BASDAI, and laboratory tests

Parameter	Category	DISQ score (Mean ± SD)	p
Diarrhea	Absent (n = 159)	5.99 ± 3.92	< 0.001
	Present (n = 15)	13.07 ± 4.32	
Abdominal pain	Absent (n = 147)	5.66 ± 3.49	< 0.001
	Present (n = 27)	11.74 ± 5.39	
Oral aphthous ulcers	Absent (n = 140)	6.21 ± 4.38	0.008
	Present (n = 34)	8.12 ± 4.37	
Perianal disease	Absent (n = 133)	6.29 ± 4.3	0.088
	Present (n = 41)	7.54 ± 4.77	
Mucus in stool	Absent (n = 167)	6.08 ± 4.06	< 0.001
	Present (n = 7)	10.2 ± 5.34	
Anemia	Absent (n = 142)	6.4 ± 4.48	0.120
	Present (n = 32)	7.38 ± 4.17	
Iron deficiency anemia	Absent (n = 155)	6.43 ± 4.47	0.067
	Present (n = 19)	7.84 ± 3.96	
BASDAI	< 4 (n = 97)	5.06 ± 3.34	< 0.001
	≥ 4 (n = 77)	8.49 ± 4.89	

*BASDAI* Bath ankylosing spondylitis disease activity index, *DISQ* Dudley inflammatory bowel symptom questionnaire

### Reliability

The Turkish version of the DISQ demonstrated acceptable internal consistency, with a Cronbach’s alpha coefficient of 0.70. Test–retest reliability was good, with an intraclass correlation coefficient (ICC) of 0.76 (two-way mixed-effects model, absolute agreement; 95% CI: 0.51–0.82; p < 0.001), indicating satisfactory temporal stability.

## Discussion

Spondyloarthritides (SpA) comprise a group of chronic inflammatory diseases sharing common pathophysiological, genetic, clinical, and radiological features. Inflammatory bowel disease (IBD) is a well-recognized extra-articular manifestation of SpA, occurring alongside axial and peripheral joint involvement. Although ileocolitis has been consistently demonstrated in endoscopic studies of patients with SpA, these intestinal lesions have traditionally been considered clinically silent in the majority of cases. Consequently, bowel symptoms in SpA have received relatively little systematic attention, despite their potential clinical relevance. This highlights an unmet need for reliable instruments capable of quantifying bowel symptoms in SpA populations.

To date, no method specifically designed to quantify bowel symptom burden in SpA has been widely implemented. In the present study, we confirmed that the Turkish version of the Dudley Inflammatory Bowel Symptom Questionnaire (DISQ) is a valid and reliable tool for assessing bowel symptoms in patients with ax-SpA, demonstrating good internal consistency and reliability.

It has been reported that approximately 5–10% of patients with AS develop overt IBD, whereas up to 60% exhibit clinically silent macroscopic and/or microscopic intestinal inflammation [9, 21]. In response to this high prevalence of subclinical gut involvement, several non-invasive tools have been developed to detect or predict intestinal inflammation. Among these, the DISQ has been proposed as a symptom-based assessment instrument [22]. Stebbings et al. previously validated the DISQ and demonstrated a significant association with disease activity as measured by BASDAI [16]. Previous research has also shown that higher disease activity in ax-SpA, as measured by BASDAI, is independently associated with microscopic intestinal inflammation [23]. In line with these findings, we observed a moderate and statistically significant correlation between DISQ scores and BASDAI in our cohort, as well as a significant correlation with BASFI, supporting an association between bowel symptom burden and musculoskeletal disease impact.

Nevertheless, it should be acknowledged that intestinal symptoms and objective markers of gut inflammation do not always parallel musculoskeletal disease activity in SpA, underscoring the complexity of the gut–joint axis [24, 25]. Indeed, several studies have shown that objective measures of intestinal inflammation, such as fecal calprotectin or histologic findings, may not consistently correlate with clinical disease activity or structural progression. In this regard, patient-reported symptom instruments such as the DISQ may provide complementary information to biological markers by capturing the subjective and clinically meaningful burden of gastrointestinal symptoms that may not always parallel objective markers of intestinal inflammation [26, 27].

In addition to disease activity measures, we evaluated the relationship between DISQ scores and health-related quality of life using the SF-36. DISQ scores were negatively correlated with all SF-36 subdomains, indicating that increased bowel symptom burden is associated with impaired physical and mental well-being. This finding is consistent with previous work demonstrating that generic and disease-specific quality-of-life instruments offer complementary insights, with generic tools such as the SF-36 capturing broader functional and psychosocial impacts beyond disease-specific activity indices [28].

Consistent with these findings, the ≥ 11 cut-off proposed in the original DISQ validation study was defined as indicative of clinically relevant bowel symptoms affecting quality of life. In our cohort, higher DISQ scores were significantly associated with greater disease activity (BASDAI), worse functional status (BASFI), and poorer health-related quality of life (SF-36), supporting the clinical relevance of bowel symptom burden in patients with axial spondyloarthritis. Thus, this threshold may help identify patients with clinically relevant gastrointestinal symptoms who may benefit from further evaluation. However, this cut-off should be interpreted cautiously, as gastrointestinal symptoms in ax-SpA may arise from causes other than inflammatory bowel disease (e.g., irritable bowel syndrome or medication-related effects), potentially leading to false positive results, while subclinical intestinal inflammation may occur in asymptomatic patients, resulting in false negative findings.

The strengths of our study include the relatively large patient cohort compared with existing validation studies, the limited number of comparable investigations in the literature, and the comprehensive evaluation of bowel symptoms in relation to disease activity, functional status, and quality of life. These aspects enhance the clinical relevance of our findings.

Several limitations should be acknowledged. First, patients with confirmed inflammatory bowel disease were excluded from the study population, unlike in the original study by Stebbings et al. Consequently, the questionnaire's ability to discriminate between ax-SpA patients with and without established IBD could not be directly assessed. However, the primary aim of this study was to evaluate the reliability and validity of the Turkish version of the DISQ in patients with axial spondyloarthritis and to assess gastrointestinal symptom burden suggestive of intestinal involvement within this population. Second, the DISQ is a symptom-based questionnaire and should not be interpreted as a diagnostic tool for intestinal inflammation or inflammatory bowel disease. Because endoscopic evaluation or objective biomarkers of intestinal inflammation were not assessed in the present study. To further investigate the relationship between DISQ scores and objective intestinal inflammation, we have conducted an additional study evaluating DISQ in conjunction with fecal calprotectin levels in patients with axial spondyloarthritis, and the analysis of these data is currently ongoing. Third, the discriminant validity analysis relied on gastrointestinal symptoms that overlap with DISQ items, potentially introducing a degree of circularity. Future validation studies incorporating independent external measures such as fecal calprotectin, endoscopic findings, or clinical referral outcomes would further strengthen the evaluation of the questionnaire’s discriminant validity and help clarify the relationship between DISQ scores and objective intestinal inflammation.

In conclusion, the Turkish version of the DISQ demonstrated acceptable validity and reliability for assessing gastrointestinal symptom burden in patients with axial spondyloarthritis. Although it should not be interpreted as a surrogate for objective intestinal inflammation, the questionnaire may serve as a practical clinical tool to identify patients with bowel symptoms who may benefit from further gastroenterological evaluation and multidisciplinary assessment. Future studies incorporating objective markers of intestinal inflammation are warranted to establish clinically meaningful cut-off values and to clarify the role of the DISQ as a screening and monitoring instrument in patients with spondyloarthritis.

## Supplementary Information

Below is the link to the electronic supplementary material.

Supplementary file1 (DOCX 33 KB)

Supplementary file2 (DOCX 19 KB)

## Data Availability

The data are available upon reasonable request.
