# Supplementary material for: Validation of the Dudley inflammatory bowel symptom questionnaire for assessing gastrointestinal symptom burden in axial spondyloarthritis
Source: Rheumatol Int. 2026 Apr 9;46(4):76. doi: 10.1007/s00296-026-06108-1 (PMC13061768; doi:10.1007/s00296-026-06108-1)
Supplement: Supplementary file 1 — Supplementary file1 (DOCX 33 KB) [file 296_2026_6108_MOESM1_ESM.docx]

Supplementary Table 1. Turkish version of the Dudley Inflammatory Bowel Symptom Questionnaire (DISQ) used in the present study

| 1. Dışkılama sıklığı | 0 | 1 | 2 | 3 | 4 |
| --- | --- | --- | --- | --- | --- |
| 2. İshal | 0 | 1 | 2 | 3 | 4 |
| 3. Dışkıda kan | 0 | 1 | 2 | 3 | 4 |
| 4. Gece dışkılama için uyanma | 0 | 1 | 2 | 3 | 4 |
| 5. Acil dışkılama hissi | 0 | 1 | 2 | 3 | 4 |
| 6. Dışkılamadan sonra devam eden şiddetli ve ısrarcı dışkılama hissi | 0 | 1 | 2 | 3 | 4 |
| 7. Ikınmayla dışkılamaya devamlı ihtiyaç duyma | 0 | 1 | 2 | 3 | 4 |
| 8. Kazara iç çamaşırına dışkılama | 0 | 1 | 2 | 3 | 4 |
| 9. Makatta kaşıntı ve ağrı | 0 | 1 | 2 | 3 | 4 |
| 10. Gaz kaçırma | 0 | 1 | 2 | 3 | 4 |
| 11. Karında kramp veya ağrı | 0 | 1 | 2 | 3 | 4 |
| 12. İştah kaybı | 0 | 1 | 2 | 3 | 4 |
| 13. Bulantı | 0 | 1 | 2 | 3 | 4 |
| 14. Halsizlik | 0 | 1 | 2 | 3 | 4 |
| 15. Ateş | 0 | 1 | 2 | 3 | 4 |

(0 = none/never; 1 = mild/sometimes; 2 = moderate; 3 = severe; 4 = very severe)
