# Supplementary material for: Validation of the Dudley inflammatory bowel symptom questionnaire for assessing gastrointestinal symptom burden in axial spondyloarthritis
Source: Rheumatol Int. 2026 Apr 9;46(4):76. doi: 10.1007/s00296-026-06108-1 (PMC13061768; doi:10.1007/s00296-026-06108-1)
Supplement: Supplementary file 2 — Supplementary file2 (DOCX 19 KB) [file 296_2026_6108_MOESM2_ESM.docx]

### Supplementary Table 2. Demographic, Clinical, Imaging and Laboratory Characteristics of Patients

|  | **Mean ± SD** or **n (%)** |
| --- | --- |
| **Number of patients** | 174 |
| **Age (years)** | 44.5 ± 10.4 |
| **Age at disease onset (years)** | 29.8 ± 9.02 |
| **Age at diagnosis (years)** | 34.3 ± 10.2 |
| **Disease duration (months)** | 175.4 ± 104.3 |
| **Gender** (Male) | 118 (67.8%) |
| **Inflammatory back pain** | 174 (100%) |
| **Arthritis** | 28 (16.1%) |
| **Dactylitis** | 7 (4%) |
| **Enthesitis** | 105 (60.3%) |
| **Uveitis** | 27 (15.5%) |
| **Psoriasis** | 7 (4%) |
| **Inflammatory bowel disease (IBD)** | 0 (0%) |
| **Family history of SpA** | 50 (28.7%) |
| **Response to NSAIDs** | 157 (90.2%) |
| **MRI-confirmed sacroiliitis (n=135)** | 127 (94.1%) |
| **Sacroiliitis on radiography (n=167)** |  |
| **Grade 0** | 10 (6%) |
| **Grade 1** | 7 (4.2%) |
| **Grade 2** | 42 (25.1%) |
| **Grade 3** | 52 (31.1%) |
| **Grade 4** | 56 (33.5%) |
| **Syndesmophyte on radiography (n=167)** | 57 (34.1%) |
| **Enthesitis on radiography (n=128)** | 66 (51.6%) |
| **HLA-B27 positivity (n=154)** | 107 (69.5%) |
| **Leukocytes (µL)** | 8147.8 ± 1902.1 |
| **Neutrophils (µL)** | 4272.6 ± 1413.4 |
| **Lymphocytes (µL)** | 2918.5 ± 923.7 |
| **Lymphocytes (%)** | 35.50 ± 9.3 |
| **Hemoglobin (g/dL)** | 14.04 ± 1.7 |
| **Hematocrit (%)** | 42.5 ± 5.4 |
| **Mean corpuscular volume (MCV, fL)** | 85.84 ± 4.6 |
| **Red cell distribution width (RDW, fL)** | 40.2 ± 4.2 |
| **Platelets (µL)** | 281,431 ± 72,344.4 |
| **Mean platelet volume (MPV, fL)** | 10.03 ± 0.93 |
| **Iron (µg/dL)** | 74.8 ± 32.2 |
| **Total iron-binding capacity (TIBC, µg/dL)** | 336.6 ± 55.2 |
| **Ferritin (µg/L)** | 77.7 ± 74.6 |
| **Vitamin B12 (µg/L, n=162)** | 310.3 ± 154.03 |
| **Erythrocyte sedimentation rate (ESR, mm/h, n=171)** | 11.8 ± 10.40 |
| **C-reactive protein (CRP, mg/L)** | 5.87 ± 9.00 |
| **Albumin (g/L, n=170)** | 44.92 ± 2.66 |
| **Globulin (g/L, n=164)** | 27.67 ± 3.91 |
| **Creatinine (mg/dL)** | 0.74 ± 0.16 |
| **Glomerular filtration rate (GFR, mL/min/1.73m²)** | 110.00 ± 80.98 |
| **AST (U/L)** | 18.78 ± 6.51 |
| **ALT (U/L)** | 23.04 ± 14.30 |

SpA, Spondyloarthritis; NSAIDs, non-steroidal anti-inflammatory drugs; MRI, Magnetic Resonance Imaging; AST, Aspartate Aminotransferase; ALT, Alanine Transaminase
